# Deficient Dopamine D_2_ Receptor Function Causes Renal Inflammation Independently of High Blood Pressure

**DOI:** 10.1371/journal.pone.0038745

**Published:** 2012-06-14

**Authors:** Yanrong Zhang, Santiago Cuevas, Laureano D. Asico, Crisanto Escano, Yu Yang, Annabelle M. Pascua, Xiaoyan Wang, John E. Jones, David Grandy, Gilbert Eisner, Pedro A. Jose, Ines Armando

**Affiliations:** 1 Division of Nephrology, Department of Medicine, School of Medicine, University of Maryland, Baltimore, Maryland, United States of America; 2 Department of Physiology and Pharmacology, Oregon Health and Science University, Portland, Oregon, United States of America; 3 Department of Medicine, Georgetown University Medical Center, Washington DC, United States of America; INSERM, France

## Abstract

Renal dopamine receptors participate in the regulation of blood pressure. Genetic factors, including polymorphisms of the dopamine D_2_ receptor gene (*DRD2)* are associated with essential hypertension, but the mechanisms of their contribution are incompletely understood. Mice lacking *Drd2* (D_2_−/−) have elevated blood pressure, increased renal expression of inflammatory factors, and renal injury. We tested the hypothesis that decreased dopamine D_2_ receptor (D_2_R) function increases vulnerability to renal inflammation independently of blood pressure, is an immediate cause of renal injury, and contributes to the subsequent development of hypertension. In D_2_−/− mice, treatment with apocynin normalized blood pressure and decreased oxidative stress, but did not affect the expression of inflammatory factors. In mouse RPTCs *Drd2* silencing increased the expression of TNFα and MCP-1, while treatment with a D_2_R agonist abolished the angiotensin II-induced increase in TNF-α and MCP-1. In uni-nephrectomized wild-type mice, selective *Drd2* silencing by subcapsular infusion of *Drd2* siRNA into the remaining kidney produced the same increase in renal cytokines/chemokines that occurs after *Drd2* deletion, increased the expression of markers of renal injury, and increased blood pressure. Moreover, in mice with two intact kidneys, short-term *Drd2* silencing in one kidney, leaving the other kidney undisturbed, induced inflammatory factors and markers of renal injury in the treated kidney without increasing blood pressure. Our results demonstrate that the impact of decreased D_2_R function on renal inflammation is a primary effect, not necessarily associated with enhanced oxidant activity, or blood pressure; renal damage is the cause, not the result, of hypertension. Deficient renal D_2_R function may be of clinical relevance since common polymorphisms of the human *DRD2* gene result in decreased D_2_R expression and function.

## Introduction

Dopamine synthesized in the kidney is necessary for the maintenance of normal blood pressure and renal function [1]. The disruption of any of the dopamine receptor subtype genes in mice produces receptor subtype-specific hypertension [2]. In particular, the hypertension in mice with disruption of the dopamine D2 receptor (*Drd2*) gene (D_2_−/−) is associated with increased production of reactive oxygen species (ROS) [3], [4].

Infiltration of inflammatory cells and oxidative stress in the kidney are involved in the development of renal injury and the induction and maintenance of hypertension [5]. Renal tubule cells produce both pro- and anti-inflammatory cytokines and chemokines [6], which are secreted across their apical and basolateral membranes [7], and contribute to the development and progression of glomerular and tubular injury. However, the factors that regulate cytokine production in these cells are incompletely understood. Dopamine and dopaminergic drugs have been shown to regulate the immune response and the inflammatory reaction [8]. Dopamine inhibits the release of IFNγ, IL-2, and IL-4 [9] and the lipopolysaccharide-stimulated production of IL-12p40 [10] in immune cells. Administration of dopamine or dopaminergic agonists *in vivo* reduces the TNFα response to endotoxin [11] and the activation of leukocytes in experimental sepsis [12]. Conversely, treatment with a dopaminergic antagonist stimulates constitutive and inducible gene expression of IL-1β, IL-6, and TNFα in macrophages [13]. In brain-dead rats, a condition that is associated with profound inflammation in end-organs, dopamine reduces renal monocyte infiltration [14], expression of IL-6, and improves renal function after transplantation [15]. Furthermore, mice with intrarenal dopamine deficiency have increased oxidative stress and infiltration of inflammatory cells [16] and decreased renal dopamine production is associated with increased detrimental effects of Ang II on renal injury [17].

The anti-inflammatory effects of dopamine and dopaminergic agonists are mediated, at least in part, by the D_2_R. D_2_Rs are expressed in lymphocytes, monocytes, neutrophils, macrophages, and other immuno-competent cells [18]. The D_2_R/D_3_R agonist, bromocriptine, inhibits lymphocyte proliferation [19] and decreases antigen-induced macrophage activation and secretion of IL-2, IL-4, and IFNγ [11]. In normal human lymphocytes, D_2_R agonists increase the secretion of anti-inflammatory cytokines by *de novo* gene expression [20]. GLC756, a novel mixed dopamine D_1_R antagonist and D_2_R agonist, inhibits the release of TNFα from activated mast cells [21].

**Figure 1:**
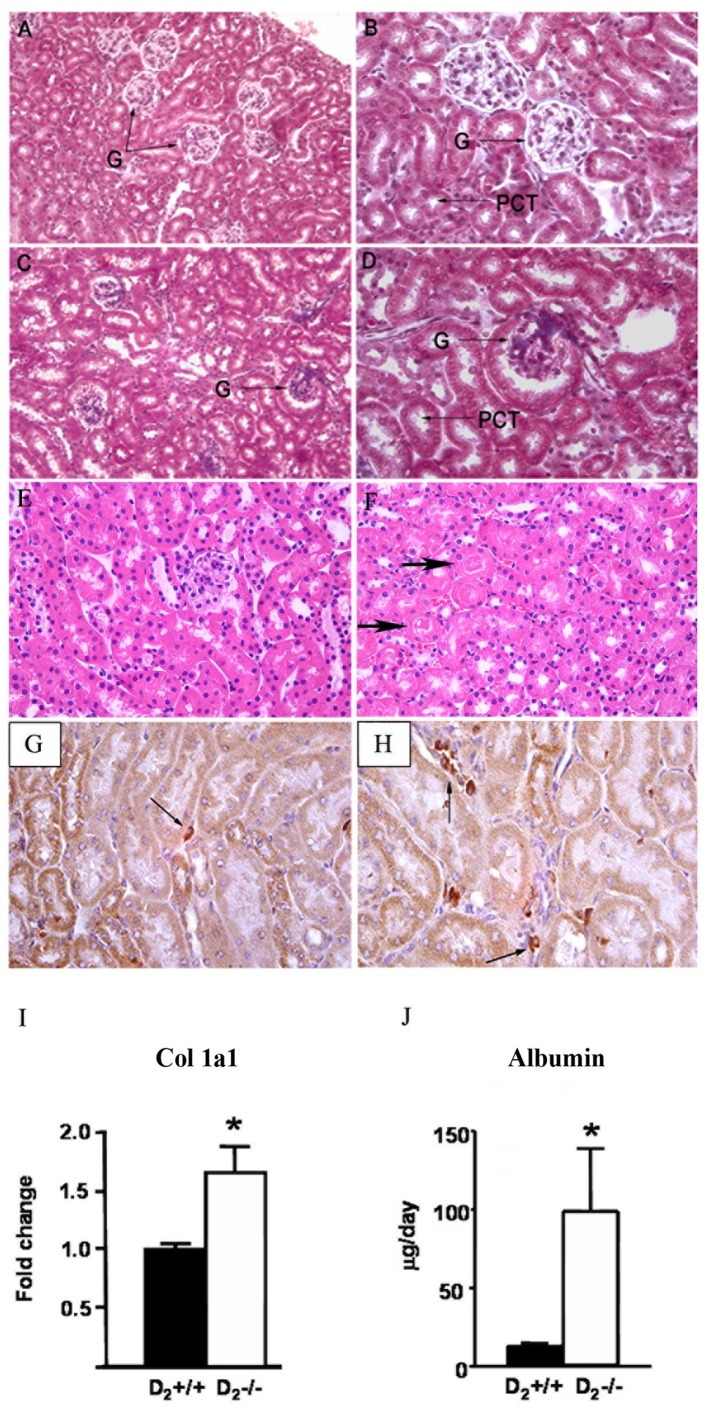
Renal inflammation and injury in D_2_−/− mice. Masson stained sections of D_2_+/+ mouse kidney (**A** and **B**) and D_2_−/− mouse kidney (**C** and **D**). H-E stained sections of D_2_+/+ mouse kidney (**E**) and D_2_−/− mouse kidney (**F**). G: glomerulus. PCT: proximal convoluted tubule. Proteinaceous casts are marked with arrows (**F**). Sections from 3 mouse kidneys per group were studied. **G** and **H**: Inflammatory cell infiltration. Kidney sections from D_2_+/+ (**G**) and D_2_−/− (**H**) mice were immunostained for the presence of macrophages and monocytes (arrows). The number of positive cells in 10 randomly selected fields was greater in D2−/− (68±3) than in D2+/+ (15±1, P<0.01) mice. Sections from 3 mouse kidneys per group were studied. **I**. Renal cortical expression of Col 1α1 mRNA determined by qRT-PCR. Results were corrected for expression of GAPDH mRNA and expressed as fold change in comparison to their expression in D_2_+/+ mice. *P<0.05 vs D_2_+/+; n = 5/group. **J**. Urinary microalbuminuria. Urine samples were collected for 24 h from mice in metabolic cages. Albumin was measured by ELISA. *P<0.04 vs. D_2_+/+; n = 5/group. Magnification: A and C: 100X; B, D, G and H: 400X; E-F: 200X.

We hypothesized that the D_2_R decreases renal inflammation and prevents renal injury by regulating the inflammatory response in renal proximal tubule cells (RPTCs). To test this hypothesis, we studied parameters of inflammation and injury in the renal cortex of D_2_−/− mice and the effect of D_2_ R silencing on the expression of inflammatory factors in mouse RPTCs. Because angiotensin (Ang) II and dopamine receptors counter-regulate each other and Ang II, via the AT_1_R, promotes inflammation and renal injury [17], [18], [22], we also determined if stimulation of D_2_R opposes the effects of Ang II in these cells. Because D_2_R deficiency increases blood pressure and oxidative stress, we studied the effects of normalizing blood pressure and decreasing oxidative stress on the renal expression of cytokines/chemokines in D_2_−/− mice. Finally, we studied renal expression of inflammatory factors and markers of renal injury in two mouse models of selective *Drd2* silencing in the kidney.

**Table 1:** Gene expression profiling of cytokines, chemokines and receptors in the kidney of D_2_+/+ and D_2_−/− mice.

Genes		Fold change
**Up-regulated**		
*Ccl2*	Chemokine (C-C motiv) ligand 2 (MCP-1)	1.87
*Ccl8*	Chemokine (C-C motiv) ligand 8 (MCP-2)	1.95
*Ccl7*	Chemokine (C-C motiv) ligand 7 (MCP-3)	2.19
*Ccl12*	Chemokine (C-C motiv) ligand 12 (MCP-4)	2.78
*Tnfα*	Tumor necrosis factor alpha	1.65
*Ltα*	Lymphotoxin α (Lta/TNF β)	1.41
*Ltβ*	Lymphotoxin β (Ltb/TNF C)	2.02
*Cxcr5*	Chemokine (C-X-C motif) receptor 5	2.19
*Ccl11*	Chemokine (C-C motif) ligand 11 (eotaxin-1)	2.68
*Ccl17*	Chemokine (C-C motif) ligand 17	2.37
*Ccl20*	Chemokine (C-C motif) ligand 20	3.09
*Ccl25*	Chemokine (C-C motif) ligand 25	2.75
*Ccr7*	Chemokine (C-C motif) receptor 7	2.61
*Cxcl9*	Chemokine (C-X-C motif) ligand 9 (MIG)	2.16
*Ccl5*	Chemokine (C-C motif) ligand 5 (RANTES)	1.59
*Ccl4*	Chemokine (C-C motif) ligand 4 (MIP-α)	1.64
*Cxcl10*	Chemokine (C-X-C motif) ligand 10	1.72
*Cxcl11*	Chemokine (C-X-C motif) ligand 11	1.71
*Il-10*	Interleukin 10	1.78
*Il-18*	Interleukin 18	2.07
*Il-5 rα*	Interleukin 5 receptor, α	3.13
**Down-regulated**		
*Ccl1*	Chemokine (C-C motif) ligand 1	−2.46
*Ccl24*	Chemokine (C-C motif) ligand 24	−2.00
*Ccr1*	Chemokine (C-C motif) receptor 1	−2.27
*Crp*	C-reactive protein, pentraxin-related	−2.49
Pf4	Platelet factor 4	−2.03
*Cxcl12*	Chemokine (C-X-C motif) ligand 12	−1.75
*Il-11*	Interleukin 11	−2.02
*Il-13*	Interleukin 13	−3.20
*Il-17B*	Interleukin 17B	−3.57
*Il-20*	Interleukin 20	−5.70
*Il-3*	Interleukin 3	−2.36
*Il-4*	Interleukin 4	−1.71
*Il-1f6*	Interleukin 1 family, member 6	−2.43
*Il-8rβ*	Interleukin 8 receptor,	−3.72
*Cd40lg*	CD40 ligand	−2.90

Fold-change was calculated by the Δ Ct method. n = 3/group.

## Methods

### D_2_ Receptor-deficient Mice

The original F2 hybrid strain (129/SvXC57BL/6J, Oregon Health Sciences University) that contained the mutated *Drd2* allele (D_2_−/−) was bred onto the C57BL/6J background for >20 generations [3]. All animal-related studies were approved by the Institutional Animal Care and Use Committee. D_2_−/− mice and wild-type littermates (D_2_+/+) were studied at 6 to 8 months of age. Mice were housed in metabolic cages for 24 h urine collection and then anesthetized for blood pressure measurement via the femoral artery, as reported previously [4]. The organs were harvested and flash-frozen. As we have reported previously [4], both systolic (121±3 (D_2_−/−) vs. 89 (D_2_+/+) mm Hg; n = 9; P<0.01) and diastolic blood pressures (87±2 (D_2_−/−) vs. 63±5 (D_2_+/+) mm Hg; n = 9; P<0.02), were increased in D_2_−/− mice, relative to D_2_+/+ littermates. A group of mice was treated for 10 days with apocynin (3 mg/kg/day, Sigma, St. Louis, MO), which inhibits NADPH oxidase activity, or vehicle, via a subcutaneously implanted osmotic mini-pump (Alzet®, Cupertino, CA). Urine collection, blood pressure measurement and tissue harvesting were performed as described above.

**Figure 2:**
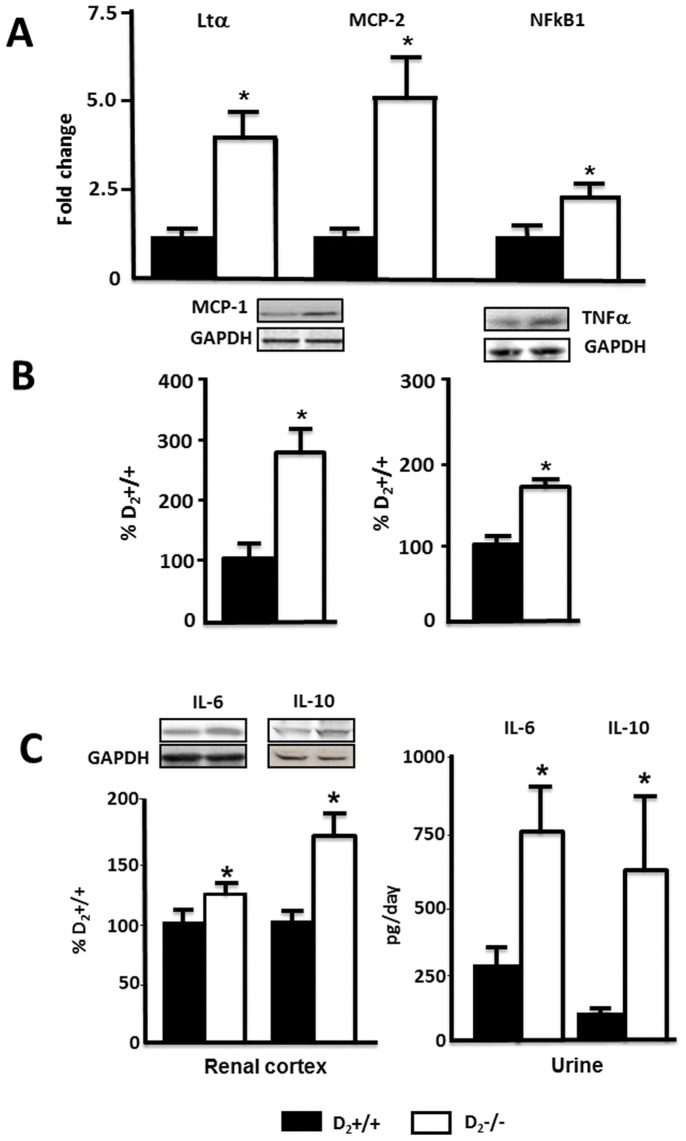
Expression of chemokines/cytokines in renal cortex and urine of D_2_−/− mice. A . Expression of Ltα, MCP-2, and NFkB1 mRNA was quantified by qRT-PCR; results were corrected for expression of GAPDH mRNA and expressed as fold change in comparison to their expression in D_2_+/+ mice. *P<0.03 vs. D_2_+/+ mice. **B**. Protein expression of MCP-1 (17 kDa) and TNFα protein (25 kDa) was semi-quantified by immunoblotting. Inset shows one set of immunoblots. Results were corrected for expression of actin and expressed as percentage of the expression in D_2_+/+ mice, *P<0.02 vs. D_2_+/+ mice, n = 5/group. **C.** Protein expression of IL-6 (25 kDa) and IL-10 (20 kDa) protein semi-quantified by immunoblotting. Results were corrected for expression of actin and expressed as percentage of the expression in D_2_+/+, * P<0.05 vs. D_2_+/+ mice, n = 5/group Urinary excretion of IL-6 and IL-10 was quantified by ELISA. *P<0.02 vs. D_2_+/+ mice, n = 5/group.

### Acute Renal Specific Down-regulation of D_2_R

Renal cortical *Drd2* was silenced by the subcapsular infusion of *Drd2*-specific siRNA via an osmotic minipump. Adult male C57BL/6J mice were uni-nephrectomized one week prior to the implantation of the minipump. For the implantation, the mice were anesthetized with pentobarbital (50 mg/kg body weight, intraperitoneally). The osmotic minipumps (100 µl; flow rate: 0.5 µl/hr for 7 days) were filled with validated *Drd2*-specific siRNA (delivery rate 3 µg/day) or non-silencing siRNA as control. The siRNAs were dissolved in an *in vivo* transfection reagent (TransIT® In Vivo Gene Delivery System, Mirus) under sterile conditions. The minipumps were fitted with a polyethylene delivery tubing (Alzet #0007701) and the tip of the tubing was inserted within the subcapsular space of the remaining kidney. Surgical glue was applied at the puncture site to hold the tubing in place and prevent extra-renal leakage. The osmotic pump was sutured to the abdominal wall to prevent excessive movement of the pump for the duration of the study.

**Table 2:** Expression of cytokines and chemokines in the heart left ventricle of D_2_+/+ and D_2_−/− mice determined by qRT-PCR.

	ΔCt D_2_+/+	ΔCt D_2_−/−	Fold change	P
*MCP-1*	5.6±0.3	6.6±0.7	0.49	NS
*MCP-2*	8.6±1.3	8.8±0.3	0.83	NS
*Tnfα*	10.5±0.5	10.7±1.8	0.86	NS
*Ltα*	11.7±1.2	12.0±0.3	0.79	NS
*Il-5 ra*	13.6±0.4	13.9±1.8	0.83	NS
*IL-11*	13.4±0.8	13.3±1.9	1.05	NS
*IL-13*	14.0±0.4	14.2±1.4	0.90	NS

Fold-change was calculated by the ΔΔCt method. Abbreviations as in Table 1. NS = not significant; n = 5/group.

Silencing of *Drd2* was also performed in mice that did not undergo unilateral nephrectomy. *Drd2*-specific siRNA was infused, as described above, under the capsule of the left kidney of C57BL/6J mice while the right kidney was left undisturbed. In both groups, blood pressure was measured, as above, before and after the 7-day siRNA infusion. Tissues were harvested after the last blood pressure determination.

### Urine Measurements

Urinary levels of IL-6 and IL-10 (SABiosciences-Qiagen, Frederick, MD) and albumin (Albuwell M, Exocell, Philadelphia, PA) were determined by ELISA, the latter using an antibody specific for murine albumin. Values were corrected for urinary creatinine.

**Figure 3:**
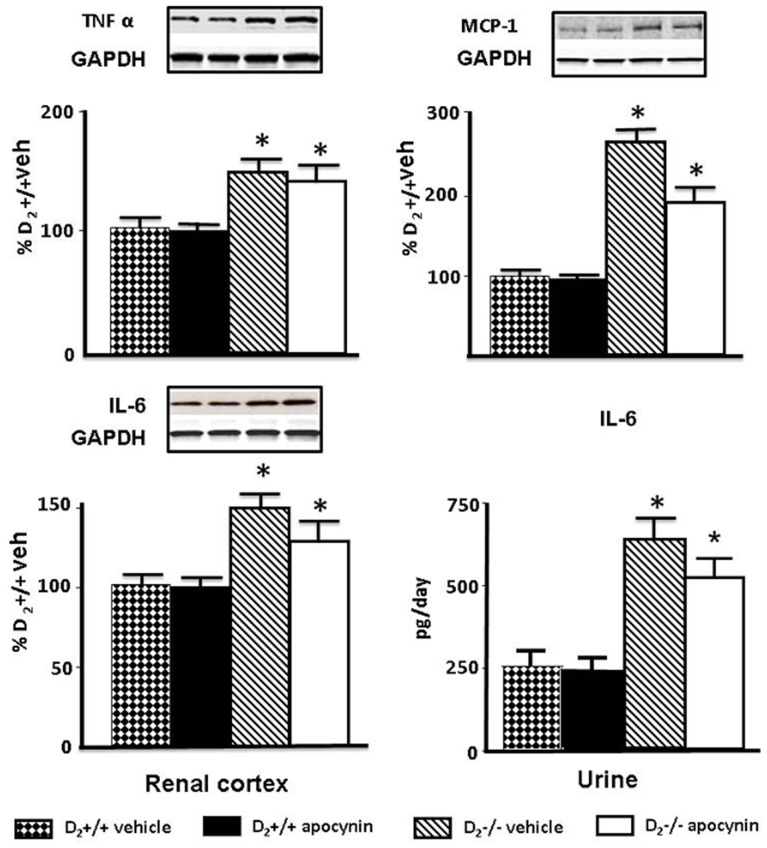
Effect of apocynin on renal cortical expression of TNFα, MCP-1, and IL-6, and urinary excretion of IL-6. Expression of TNFα (25 kDa) and MCP-1 (17 kDa) protein in renal cortex was semi-quantified by immunoblotting. Inset shows one set of immunoblots. Results were corrected for expression of GAPDH and expressed as percentage of D_2_+/+ mice treated with vehicle, *P<0.05 vs. vehicle or apocynin treated D_2_+/+; n = 5/group. Renal expression, semi-quantified by immunoblotting (25 kDa), and urinary excretion of IL-6 quantified by ELISA in 24 h urine samples. Results are expressed as percentage of D_2_+/+ mice treated with vehicle. *P<0.05 vs. vehicle or apocynin treated D_2_+/+; n = 5/group.

### Cell Culture

Undifferentiated mouse cells were cultured from progenitor kidney cells, kindly supplied by Dr. Ulrich Hopfer (Case Western Reserve University, School of Medicine), isolated from mouse embryo kidneys following the procedure described by Woost et al. [23]. Differentiated mouse RPTCs were cultured to 60–70% confluence and transfected (Hyperfect, Qiagen, Valencia, CA) with vehicle, non-silencing siRNA (30 nmol/l; All stars, Qiagen) or *Drd2* siRNA (30 nmol/l, Qiagen). Cells were studied after 72 h. For other experiments cells were cultured to 90–95% confluence, serum starved for 2 h and treated for 24 h in serum-free medium with vehicle (PBS) or 100 nmol/l Ang II in the presence or absence of 1 μmol/l quinpirole (D_2_R/D_3_R agonist), or 1 μmol/l quinpirole plus 1 μmol/l L-741,262 (D_2_R antagonist) [24].

**Figure 4:**
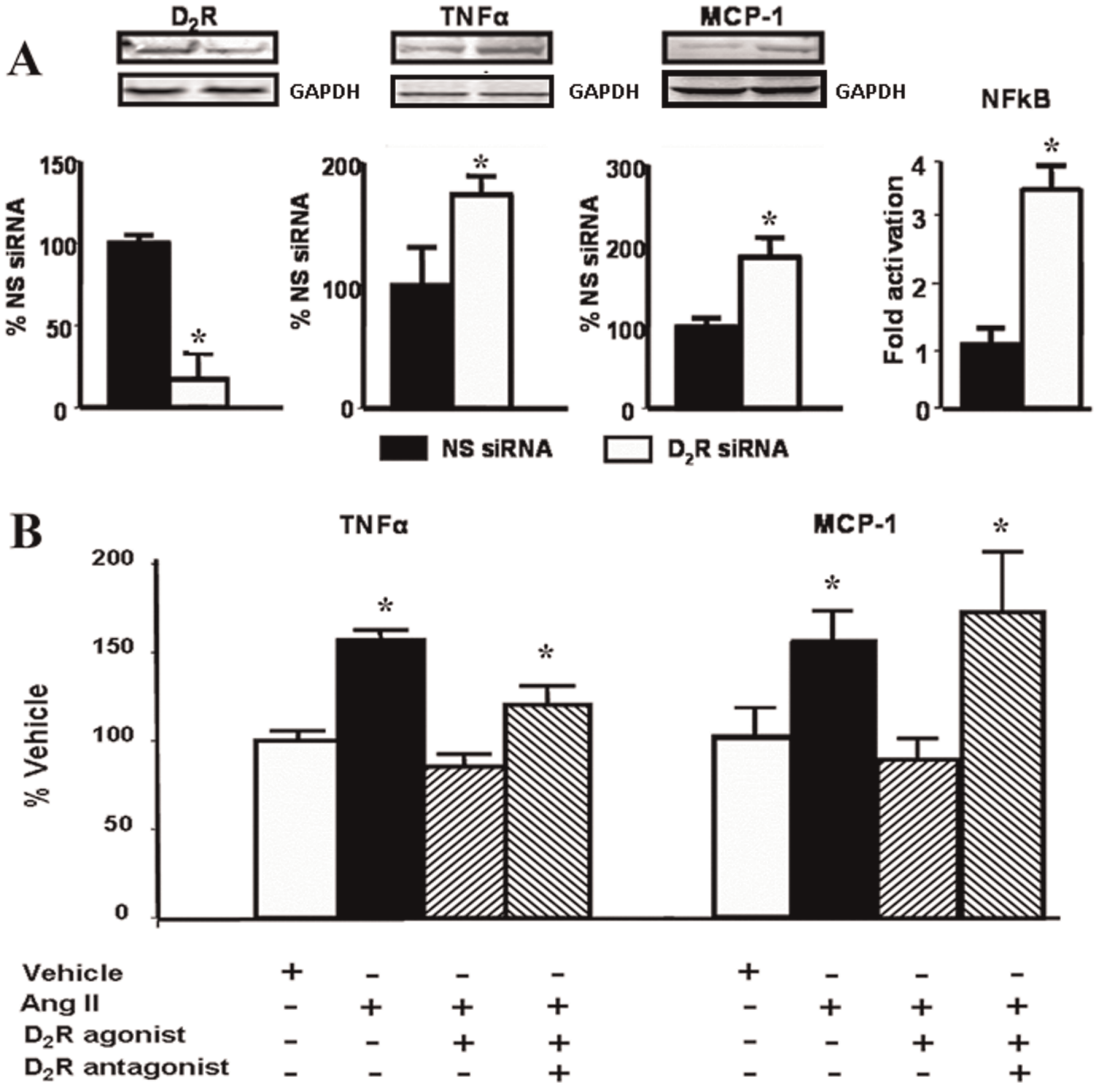
D_2_R function in moue renal proximal tubule cells A. Effect of silencing of D_2_R on the expression of pro-inflammatory cytokines/chemokines in mouse RPTCs. Cells were cultured to 60–70% confluence and transfected with non-silencing (NS siRNA) or *Drd2* siRNA. After 48 h the cells were washed and lysed. Protein expression of D_2_R (55 kDa), TNFα (25 kDa), and MCP-1(17 kDa) was semi-quantified by immunoblotting. Inset shows one set of immunoblots. NFkB activation was analyzed via the transient expression of a NFkB-luciferase reporter system by reverse transfection Results are expressed as percentage of NS siRNA or fold activation compared to NS siRNA. *P<0.05 vs. NS (non-silencing) siRNA, n = 4/group. **B**. Effects of Ang II and D_2_R stimulation on TNFα and MCP-1 in mouse RPTCs. Cells were serum starved for 2 h before treatment for 24 h in serum-free medium with vehicle (PBS) or 100 nM Ang II, in the presence or absence of 1 μM quinpirole (D_2_R/D_3_R agonist) or 1 μM quinpirole plus 1 μM L-741,262 (D_2_R antagonist). Expression of TNFα (25 kDa) and MCP-1 (17 kDa) protein was semi-quantified by immunoblotting. Inset shows one set of immunoblots. Results were corrected for actin and expressed as % of vehicle. * P<0.05 vs. vehicle; n = 6/group.

### RNA Extraction and cDNA Preparation

Kidney samples were homogenized, and total RNA was extracted with Trizol (Invitrogen, Carlsbad, CA) and further purified using the RNeasy RNA Extraction Mini kit (Qiagen). RNA samples were converted into first strand cDNA using an RT^2^ First Strand kit, following the manufacturer’s protocol (SABiosciences-Qiagen).

### Gene Expression Profiling of Inflammatory Cytokines and Receptors

Gene expression analysis was carried out in groups of four mice using an RT^2^ Profiler PCR array system (SABiosciences-Qiagen) that contained a panel of 84 genes. Real-time PCR was performed following the manufacturer’s protocol. Quality controls were all within the recommended range. Data were analyzed by the Δ Ct method [25].

**Figure 5:**
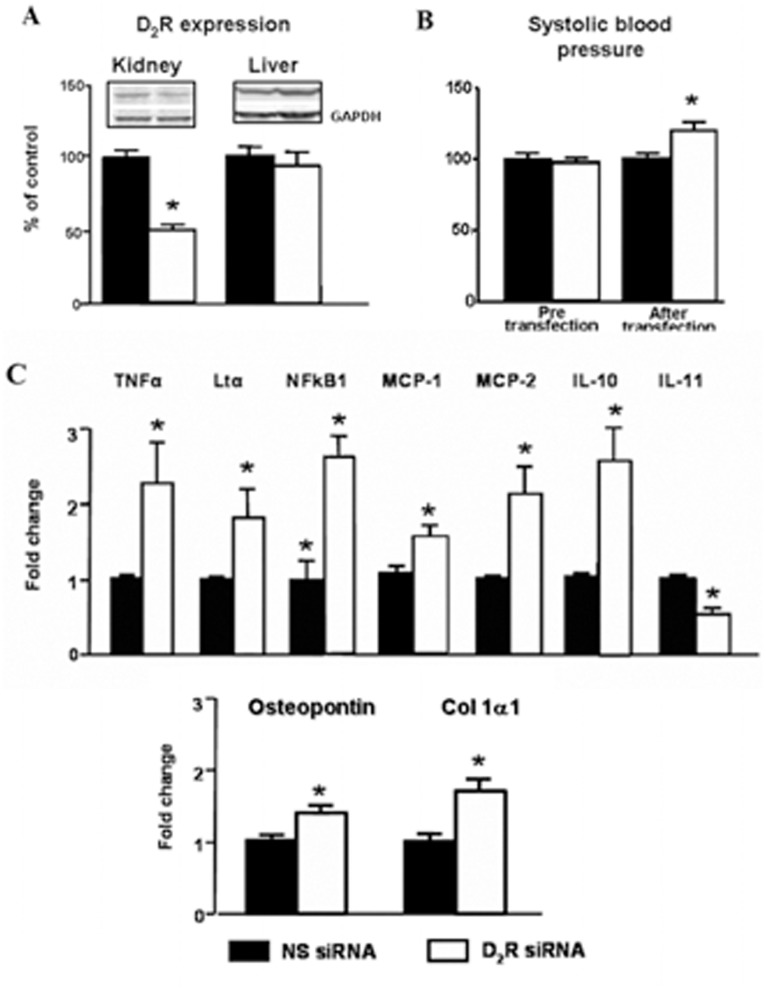
Effect of selective renal silencing of D_2_R in the remaining kidney of uni-nephrectomized mice on blood pressure and expression on inflammatory factors in the kidney and liver. Renal cortical *Drd2* was silenced by the renal subcapsular infusion for seven days of *Drd2* siRNA, via an osmotic minipump in uni-nephrectomized adult male C57BL/6J mice (see Methods). A. Expression of D_2_R protein (55 kDa band) in renal cortex and liver was semi-quantified by immunoblotting. Results were corrected for GAPDH and expressed as % of non-silencing siRNA treated kidneys. * P<0.05 vs non-silencing (NS) siRNA; n = 5/group. B. Systolic blood pressure measured under anesthesia in mice before and seven days after *Drd2* siRNA infusion. * P<0.05 vs, NS siRNA; n = 5/group. C. Renal cortical expression of TNFα, Ltα, NFkB1, MCP-1, MCP-2, IL-10, IL-11 osteopontin, and Col 1α1 mRNA was quantified by qRT-PCR, results corrected for expression of GAPDH mRNA, and expressed as fold change in comparison to their expression in mice treated with NS siRNA. *P<0.05 vs. NS; n = 5/group.

### Quantitative Real-time PCR

Quantitative gene expression was analyzed by real-time PCR, performed on an ABI Prism 7900 HT (Applied Biosystems, Foster City, CA). The assay used gene specific primers (SABiosciences-Qiagen) and SYBR Green real-time PCR detection method and was performed as described in the manufacturer’s manual. Primers used were as follows: MCP-1: PPM03151F; MCP-2: PPM03165A; TNFα: PPM03113F; Ltα: PPM03114A; IL-4: PPM03013E; IL-5αr: PPM03026E; IL-11: PPM03018E; IL-13: PPM03021A; collagen, type 1, α1 (Col 1α1): PPM-3845F; NFkB1: PPM02930E; osteopontin: PPM03648C; Actin: PPM0294A; GAPDH: PPM02946E. Data were analyzed using the Δ Δ Ct method [25].

**Figure 6:**
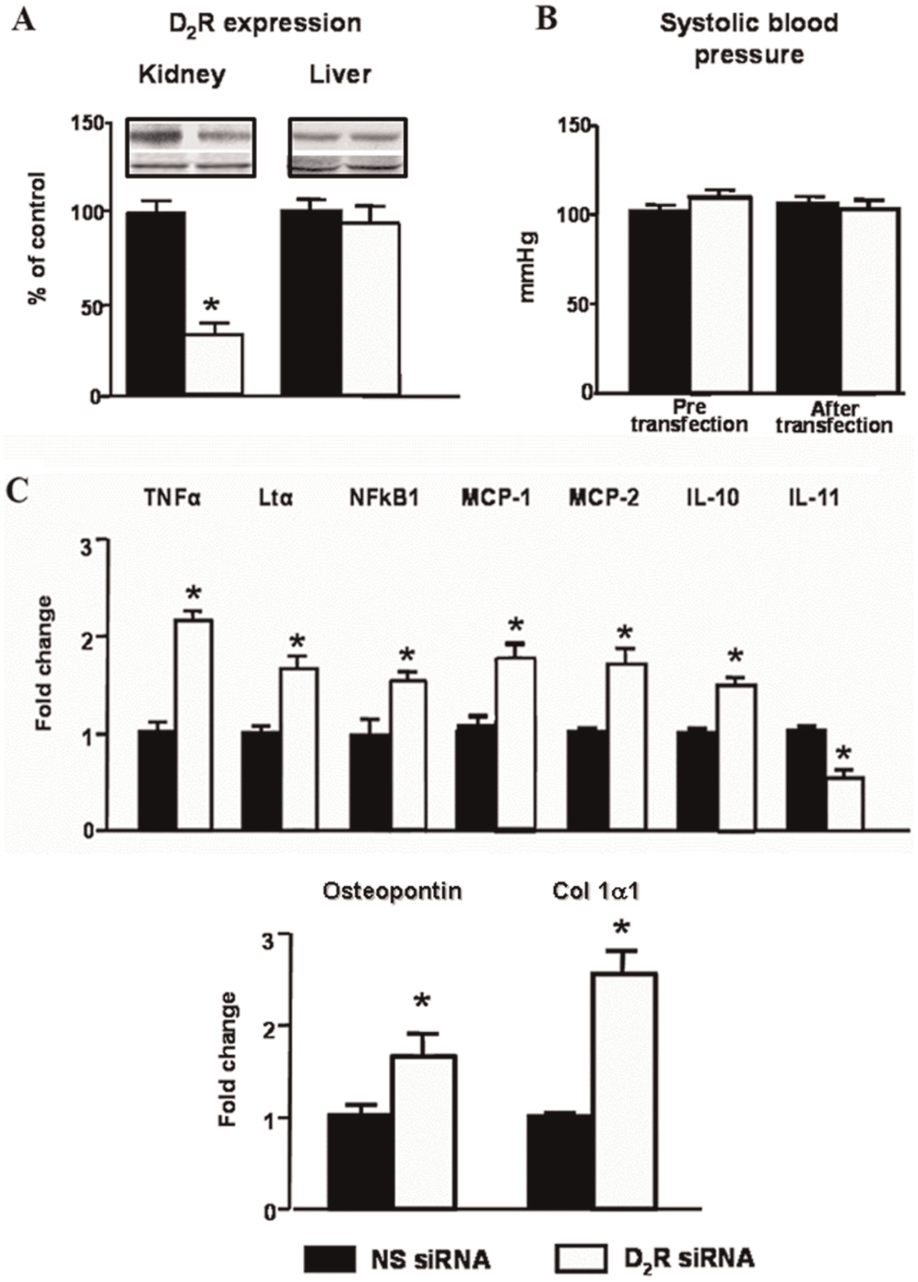
Effect of selective renal silencing of D_2_R in one kidney of mice without uni-nephrectomy on blood pressure and expression of inflammatory factors in the kidney and liver. Renal cortical D_2_R was silenced by the renal subcapsular infusion in the left kidney for seven days of *Drd2* siRNA, via an osmotic minipump in adult male C57BL/6J mice (see Methods). A. Expression of D_2_R protein (55 kDa band) in renal cortex and liver was semi-quantified by immunoblotting. Results were corrected for GAPDH and expressed as % of NS siRNA treated kidneys. * P<0.05 vs non-silencing NS siRNA; n = 4/group. B. Systolic blood pressure measured under anesthesia in mice before and seven days after *Drd2* siRNA infusion; n = 5/group. C. Renal cortical expression of TNFα, Ltα, NFkB1, MCP-1, MCP-2, IL-10, IL-11, osteopontin and collagen 1α1 mRNA was quantified by qRT-PCR, results corrected for expression of GAPDH mRNA, and expressed as fold change in comparison to their expression in mice treated with NS siRNA. *P<0.05 vs. NS; n = 5/group.

### Immunoblotting

Mouse kidney homogenates and cell lysates were subjected to immunoblotting, as reported previously [3], [4]. The primary antibodies used were rat anti-mouse TNFα (BioLegend, San Diego, CA), rabbit polyclonal MCP-1 (Millipore, Billerica, CA), rabbit polyclonal IL-6 (Abcam, Cambridge, MA); rabbit polyclonal D_2_R (Millipore), and polyclonal anti-actin (Sigma). The densitometry values were corrected by the expression of GAPDH and are shown as percentage of the mean density of the control group.

### Reporter Assay

NFkB activation was analyzed via the transient expression of an NFkB luciferase reporter system by reverse transfection (Cignal Reporter Assay, SABiosciences-Qiagen). Cells were treated with *Drd2*-specific siRNA or non-silencing siRNA, as described above. After 48 h, the cells were trypsinized and seeded for reverse transfection. The assay was performed following the manufacturer’s procedures.

### Histochemistry and Immunohistochemistry

Formalin-fixed, paraffin-embedded tissues of D_2_+/+ and D_2_−/− mice were stained with Masson trichrome to evaluate glomerular fibrosis and with hematoxylin eosin (H–E) to evaluate tubular damage. The pathological abnormalities were graded in a blinded manner. Sclerosis was defined as collapse or obliteration of the glomerular capillary tuft associated with increased hyaline matrix [26]. Glomerular sclerosis was expressed as the percentage of glomeruli showing more than 25% sclerosis.

Tissue sections were immunostained for the presence of macrophages and monocytes using a specific rat anti-mouse macrophage/monocyte monoclonal antibody (Millipore) and an avidin–biotin immunoperoxidase kit (Vectastain Elite, Vector Laboratories, Burlingame, CA). The kidneys were lightly counterstained with hematoxylin. The total number of positive cells in 10 randomly selected fields was counted.

### Statistical Analysis

Data are mean ± SEM. Comparisons between 2 groups used the Student’s t test. One-way ANOVA followed by post-hoc analysis using the Newman–Keuls multiple comparison test was used to assess significant differences among three or more groups. P<0.05 was considered statistically significant.

## Results

### Renal Injury and Inflammation Occurs in D_2_−/− Mice

Masson staining of D_2_−/− mouse kidney sections showed glomerulosclerosis and dilation of renal tubules (Fig 1C–D). H-E staining showed the presence of tubular proteinaceous casts (Figure 1F). These lesions were not observed in D_2_+/+ mice (**Figure 1A, B,E**). The percentage of glomeruli showing more than 25% sclerosis was greater in D_2_−/− than D_2_+/+ mice (35±9% vs. 5±6%, P<0.01). There were more infiltrating macrophages/monocytes in kidney sections from D_2_−/− mice (**Figure 1H**) than D_2_+/+ mice (**Figure 1G** (68±3 vs.15±1 positive cells/10 fields, P<0.01). The level of mRNA expression of Col 1α1 was about 60% higher in renal cortex of D_2_−/− than D_2_+/+ mice (**Figure 1I**). Microalbuminuria, a functional parameter of renal damage, was 9-fold higher in D_2_−/− mice than in D_2_+/+ littermates (**Figure 1J**).

### The Expression of Chemokines and Cytokines Involved in Macrophage Recruitment and Inflammation is Increased in the Renal Cortex but not in the Left Ventricle of the Heart of D_2_−/− Mice

Expression of 84 cytokines and chemokines was analyzed in the renal cortex of D_2_−/− and D_2_+/+ mice using a quantitative RT- PCR (qRT-PCR) array. Twenty one genes were up-regulated and 15 were down-regulated in D_2_−/− mice (**Table 1**). Of the genes that were up-regulated, 10 belong to the C-C subfamily of chemokines, including four of the macrophage chemoattractant group and three of the TNF superfamily. IL-10 and IL-18 genes were also up-regulated. Seven of the 15 down-regulated genes were interleukins (**Table 1**). Most of the up-regulated chemokines are inflammatory and belong to the CCL subfamily, involved in macrophage (MCP-1, MIP-1α, RANTES, MCP-2, MCP-5) and/or T cell (Eotaxin-1, TARC, MIP-3α, CCL-25) recruitment, as opposed to homeostatic [27]. Some of the chemokines, belonging to the CXCL superfamily that attract neutrophils, were also up-regulated (MIG, IP-10, I-TAC) [28]. Three of the four members of the TNF superfamily of inflammatory cytokines were up-regulated, namely TNFα, lymphotoxin-α (Ltα), and lymphotoxin-β (TNFβ). CD40L, the other member of the superfamily included in the array, was decreased. In contrast to the increased expression of pro-inflammatory chemokines, several anti-inflammatory interleukins (IL-4, IL-11, IL13, and IL-17B which stimulates IL-11) were decreased, except for IL-10 which was increased (**Table 1**).

Further experiments were focused on the TNF and MCP families and on IL-6 and IL-10, both of which are downstream TNFα, and on NF**k**B, which is activated and increased by TNFα transcription [29], [30]. IL-6 is involved in the development of renal inflammation and injury [31], and IL-10 has potent anti-inflammatory properties, repressing the expression of TNFα, IL-6, and IL-1 [32]. We also quantified the expression of p50, the DNA binding subunit of NF**k**B protein complex, a parameter of NF**k**B activation [33]. Increased renal cortex expression of Ltα, MCP-2, and NF**k**B1 (p50) in D_2_−/− mice was confirmed by qRT-PCR and found to be four-, five-, and two -fold higher, respectively, than in D_2_+/+ (**Figure 2A**). Increased protein expression of MCP-1 (270±30 vs 100±15%) and TNFα (163±7 vs 100±3%) was confirmed by western blot (**Figure 2B**). Protein expressions of IL-6 and IL-10 in renal cortex were also increased by about 30% and 60% respectively, and urinary excretion of IL-6 was about three-fold higher while that of IL-10 was about five-fold higher in D_2_−/− than in D_2_+/+ mice (**Figure 2C**). Decreased renal cortical mRNA expression of IL-4, IL-11, and IL-13 was also confirmed by qRT-PCR (data not shown).

The gene expression of chemokines/cytokines in the heart left ventricle was also determined by qRT-PCR. The expressions of MCP-1, MCP-2, TNFα, and Ltα, as well as IL-11, IL-13, and IL-5 receptor α, were similar in D_2_−/− and D_2_+/+ mice (**Table 2**). This indicated that renal alterations in pro- and anti-inflammatory factors in D_2_−/− mice were organ specific and not caused by systemic perturbations.

### Decreasing Blood Pressure and ROS does not Normalize the Expression of Inflammatory Factors in Renal Cortex of D_2_−/− Mice

Treatment with apocynin decreased systolic blood pressure in D_2_−/− mice (vehicle: 121±5; apocynin: 96±2 mm Hg; n = 5; P<0.05) but not in D_2_+/+ mice (vehicle: 98±3; apocynin 95±5 mmHg; n = 5). Apocynin also decreased the urinary excretion of the oxidative stress marker 8-isoprostane in D_2_−/− mice (vehicle: 3166±456; apocynin: 1874±553 pg/mg creatinine; n = 5, P<0.04) to levels similar to those in wild-type mice (vehicle: 1344±365; apocynin: 1542±280 pg/mg creatinine; n = 5). Treatment with apocynin, however, did not normalize the expression of TNFα, MCP-1, or IL-6 in D_2_−/− mice. TNFα expression in renal cortex was higher in vehicle-treated D_2_−/− than vehicle- treated D_2_+/+ mice; apocynin had no effect on TNFα expression in D_2_+/+ or D_2_−/− mice. MCP-1 protein expression was also higher in vehicle-treated D_2_−/− than in vehicle-treated D_2_+/+ mice; apocynin had no effect on MCP-1 expression in D_2_+/+ mice but decreased it in D_2_−/− mice although not to the level observed in D_2_+/+ mice (**Figure 3** **)**. Renal cortical IL-6 protein expression and urinary excretion of IL-6 were also higher in vehicle-treated D_2_−/− than in vehicle-treated D_2_+/+ mice; apocynin had no effect on IL-6 in D_2_+/+ mice but modestly decreased its levels in D_2_−/− mice although they remained higher than D_2_+/+ mice (**Figure 3**).

### *Drd2* Silencing in Mouse RPTCs Results in Increased NFκB Transcriptional Activity and TNFα and MCP-1 Expression

Mouse RPTCs in culture endogenously express D_2_R, TNFα, and MCP-1. Forty-eight hour-treatment with *Drd2* siRNA decreased D_2_R protein expression by about 85%. The treatment increased NF**k**B transcriptional activity (3.5-fold) and about two-fold the expression of both TNFα, and MCP-1 which are downstream of NF**k**B (**Figure 4A**).

### Stimulation of D_2_R Counteracts the Effects of Ang II in Mouse RPTCs

Treatment with Ang II (100 nmol/l) increased the expression of TNFα by about 50% and that of MCP-1 about 60% in mouse RPTCs. Treatment with quinpirole (1 μmol/l), a D_2_R/D_3_R agonist, prevented the stimulatory effect of Ang II on the expression of TNFα and MCP-1. The effect of quinpirole was blocked by the addition of L-741,262, a selective D_2_R antagonist (**Figure 4B**).

### Renal Specific *Drd2* Down-regulation Recapitulates the Effects of Germline *Drd2* Knockout on Inflammatory Factors Independently of Changes in Blood Pressure

To determine further the role of D_2_R in the renal inflammatory reaction, we acutely and selectively silenced renal *Drd2s* in mice in order to avoid the confounding effects of systemic D_2_R deletion. Infusion of *Drd2* siRNA for seven days in uni-nephrectomized mice decreased renal cortical expression of D_2_R by 50% but did not affect the expression of the receptor in the liver, indicating renal selectivity of the down-regulation (**Figure 5A**). As with systemic *Drd2* deletion, treatment with *Drd2* siRNA increased systolic blood pressure by about 20 mmHg (**Figure 5B**), an increase of the same magnitude of that observed in mice with systemic *Drd2* deletion [3], [4]. This highlights the role of D_2_R in the regulation of blood pressure via the kidney. Subcapsular renal *Drd2* silencing in uni-nephrectomized mice increased renal cortical mRNA expression of TNFα, Ltα, NF**k**B1, MCP-2 and IL-10, and simultaneously decreased the expression of IL-11. These results are similar to those found in mice with systemic *Drd2* deletion, confirming the role of renal D_2_R in the regulation of the expression of inflammatory factors. Furthermore, the expression of osteopontin and Col 1α1, markers of tissue damage [34], was also increased in the kidneys with silenced D_2_Rs (**Figure 5C**).

In order to eliminate the confounding effect of uni-nephrectomy and the increase in blood pressure in the above experiments, we also studied the effect of chronic unilateral renal subcapsular infusion of *Drd2* siRNA in mice with two intact kidneys. Selective down-regulation of *Drd2* in one kidney **(** **Figure 6A**) had no effect on systolic blood pressure (**Figure 6B**), suggesting that the intact kidney, in the short-term, is able to compensate for the effects of decreased *Drd2* expression in the treated kidney. The mRNA expression of TNFα, Ltα, NFκB1, MCP-1 and MCP-2 was increased in the treated kidney to the same extent as in treated uni-nephrectomized mice; NF**k**B1 and IL-10 were increased but to a lesser extent than in uni-nephrectomized mice. The mRNA expression of IL-11 was similarly decreased. In contrast the expression of the injury markers osteopontin and Col 1α1 was increased to a greater extent than in infused remnant kidney of uni-nephrectomized mice (**Figure 6C**).

## Discussion

Our results show increased renal expression of pro-inflammatory and decreased expression of anti-inflammatory cytokines/chemokines, as well as histological and functional evidence of renal inflammation and injury in mice lacking D_2_Rs. These alterations are renal-specific and are mimicked in mouse RPTCs in which the *Drd2* is silenced. Moreover, selective unilateral renal D_2_R down-regulation in mice with two kidneys, in the absence of elevated blood pressure, reproduced the alterations in inflammatory factors and renal injury observed in D_2_−/− mice. Thus, our findings indicate that D_2_Rs in the kidney have a direct and significant role in regulating the mechanisms involved in the development of renal inflammation and injury, as well as in blood pressure control.

Chemokines that play an essential role in the direct migration of various types of immune cells were up-regulated in kidneys of D_2_−/− mice, *Drd2*-silenced kidneys and RPTCs. In several models of renal injury, MCP-1 and RANTES are expressed in damaged renal tissues and precede the recruitment of inflammatory cells that is a characteristic of many kidney diseases [7]. The infiltrating cells mediate the initiation and progression of injury by direct cytotoxicity, secretion of pro-inflammatory cytokines, and the induction of other pro-inflammatory mediators in renal tubule cells.

The increased gene transcription/protein expression of inflammatory factors with Drd2 silencing may be caused by decreased D_2_R-dependent inhibition leading to increased production of TNFα, a major regulator of cytokine/chemokine expression. Experimental and clinical studies have demonstrated the role of TNFα as a mediator of inflammatory tissue damage in the pathogenesis of acute and chronic renal disease. TNFα is released from renal cells in response to injury and induces glomerular fibrin deposition, cellular infiltration, and vasoconstriction [35] but causes marked natriuresis [36]. TNFα stimulation increases the expression of IL-6, IL-10, and MCP-1 [22]. In immune cells, TNFα production is decreased by dopamine and D_2_R agonists [21] and in adrenal cortical cells, dopamine, through the D_2_R, inhibits basal and secretagogue-stimulated TNFα. Our results in mouse RPTCs showing increased basal TNFα expression in response to *Drd2* silencing and inhibition of Ang II-induced TNFα stimulation by D_2_R activation, indicate that in RPTCs the D_2_R negatively regulates both basal and Ang II-stimulated TNFα production.

TNFα and other members of the TNF superfamily regulate the expression of a large number of cytokines and chemokines by several mechanisms [37], one of which is the activation and nuclear translocation of NF**k**B [38]. NFκB, which is activated by TNFα, mediates the inflammatory response to TNFα, IL-1β, and other inflammatory factors in renal cells [33]. In turn, the transcription of TNFα and TNF superfamily members is increased by NF**k**B activation, generating a positive-feedback loop of activation [39]. Our data show that deficient D_2_R expression results in NF**k**B activation, as indicated by the increased renal expression of NF**k**B1 (p50) and NF**k**B transcriptional activity in mouse RPTCs. NF**k**B has been implicated as a factor in diabetic nephropathy [40]. Because the D_2_R has been shown to positively regulate NF**k**B activation in neural-derived cell lines [41], [42] it is likely that the negative regulation observed in the current studies is mediated by its direct effects on TNFα expression and function. Most of the down-regulated cytokines in the renal cortex of D_2_−/− mice are Th2-type cytokines (e.g., IL-4 and IL-13); the transcription of these cytokines is mainly dependent on factors other than TNFα or NF**k**B [43] and is negatively regulated by Th1-type cytokines [44].

The hypertension noted in D_2_−/− mice is at least partially related to increased renal production of ROS [4]. To evaluate the potentially confounding effect of high blood pressure and ROS on renal inflammation, we treated D_2_−/− mice with apocynin, which normalized both blood pressure and ROS production [4] as it does in several experimental models of hypertension [45]. Apocynin had no significant effect on the expression of TNFα, and IL-6, although it decreased MCP-1 expression. These results suggest that, in D_2_−/− mice, high blood pressure or increased ROS may contribute but neither is the major cause of the increased expression of pro-inflammatory factors. However, an effect of persistent inflammation due to preexisting hypertension cannot be ruled out.

The selective unilateral renal silencing of D_2_R for seven days, in mice with two kidneys, did not increase blood pressure but nonetheless increased renal expression of pro-inflammatory chemokines/cytokines and decreased expression of the anti-inflammatory, IL-11. This indicates that hypertension, per se, is not necessary for the development of renal inflammation but may be a contributing factor. Moreover, the expression of the anti-inflammatory, IL-10, was increased, indicating some compensatory feed-back mechanism. Nevertheless, our results show that impaired D_2_R function (due to decreased D_2_R expression) results in a defective balance of pro-inflammatory and anti-inflammatory factors that contribute to renal inflammation and injury.

As mentioned above, intrarenal dopamine buffers the deleterious effects of Ang II on renal inflammation and injury [16], [17]. Our results suggest that these effects are mediated by the D_2_R. Infusion of Ang II in rats increases TNFα production in renal glomerular endothelial cells, tubules, and vessels, and enhances expression of MCP-1 [22]. Stimulation of the D_2_R reversed the increased expression of TNFα and MCP-1 elicited by Ang II in mouse RPTCs, indicating that D_2_R may counterbalance the damaging effect of Ang II in the kidney.

The current studies contribute to the understanding of the mechanisms that cause the development of renal inflammation, as well as the development and maintenance of hypertension [5] and suggest that decreased D_2_R function may play a significant role in these processes. Deficient renal D_2_R function may be of clinical relevance since polymorphisms of the *Drd2* gene, that are commonly observed in humans, result in decreased D_2_R expression and function as a consequence of decreased D_2_R mRNA stability and decreased synthesis of the receptor or decreased receptor affinity [46]–[50]. Some of the D_2_R polymorphisms are associated with elevated blood pressure and essential hypertension [51]–[53]. Moreover, a recent study in an Asian Indian population with type 2 diabetes found that a D_2_R polymorphism, resulting in decreased expression of the receptor, confers susceptibility to chronic diabetic nephropathy [54]. Further studies are needed to establish the role of D_2_R polymorphisms in conferring susceptibility to chronic renal disease and to determine whether or not modulation of renal D_2_R function may be an option in the treatment of hypertension and renal injury.
